# Endodontic management of a maxillary second premolar with an S-shaped root canal

**DOI:** 10.4103/0972-0707.48842

**Published:** 2008

**Authors:** Joseph Reuben, Natanasabapathy Velmurugan, Santhanam Vasanthi, Priya, Vijayalakshmi

**Affiliations:** Department of Conservative Dentistry and Endodontics, Meenakshi Ammal Dental College, Chennai, India

**Keywords:** Bayonet shaped canal, double-flare technique, S-shaped canals

## Abstract

Complex and unusual root canal morphology is an often occurring phenomenon. Understanding the unusual root canal morphology contributes to success in endodontic treatment. One such variant root canal morphology is the ‘S’ shaped or bayonet shaped root canal. This case report discusses endodontic treatment of a maxillary second premolar with an ‘S’ shaped root canal.

## INTRODUCTION

Variations in root canal system occur so often that it is considered a normally occurring phenomenon. Endodontic therapy will be successful only when a thorough disinfection of the entire root canal system is achieved. Understanding these unusual root canal morphology will contribute to success in endodontics.

According to Vertucci,[1–3] maxillary premolars are the teeth with the maximum anatomic variations. One such variation that occurs often in the maxillary premolars is the ‘S’ shaped or bayonet shaped root canal. The ‘S’ shaped root canals have also been reported in maxillary laterals, maxillary canines and mandibular molars.

This report discusses the endodontic management of an ‘S’ shaped root canal.

## CASE REPORT

A 34-year-old female patient was referred to the Department of Conservative Dentistry and Endodontics, for the management of a right maxillary second premolar. On clinical examination, tooth 15 had a large carious lesion on the disto-proximal aspect. The patient gave a history of severe pain for the past three days. Radiographic examination of the tooth revealed radiolucency in the disto-proximal aspect, very close to the pulp space. The roots were doubly curved (Bayonet or ‘S’ shaped). From the clinical and radiographic findings, a diagnosis of irreversible pulpitis was made in relation to 15. Hence, endodontic treatment was initiated in 15.

**Figure 1:**
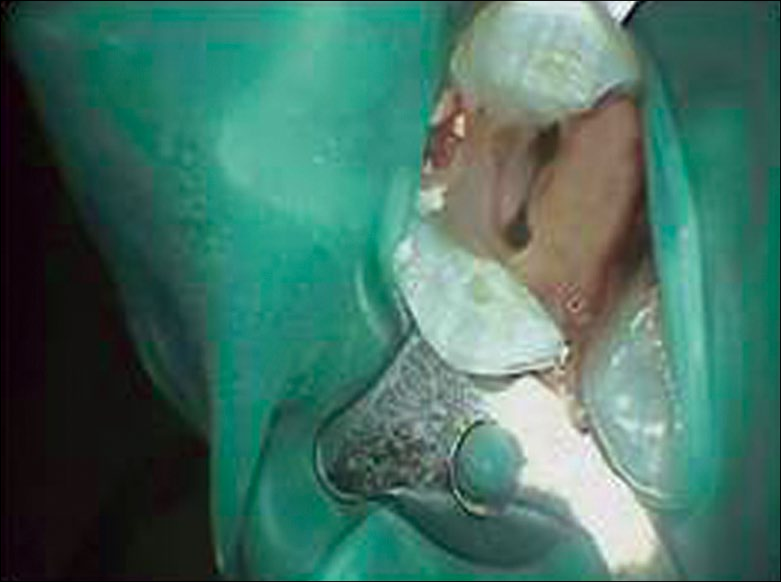
Access opening

The right upper maxillary premolar was anaesthetized with 2% lignocaine and access was opened with a No: 2 round bur, under rubber dam isolation [Figure 1]. The completed access preparation was oval in outline, in the bucco-palatal direction.

**Figure 2:**
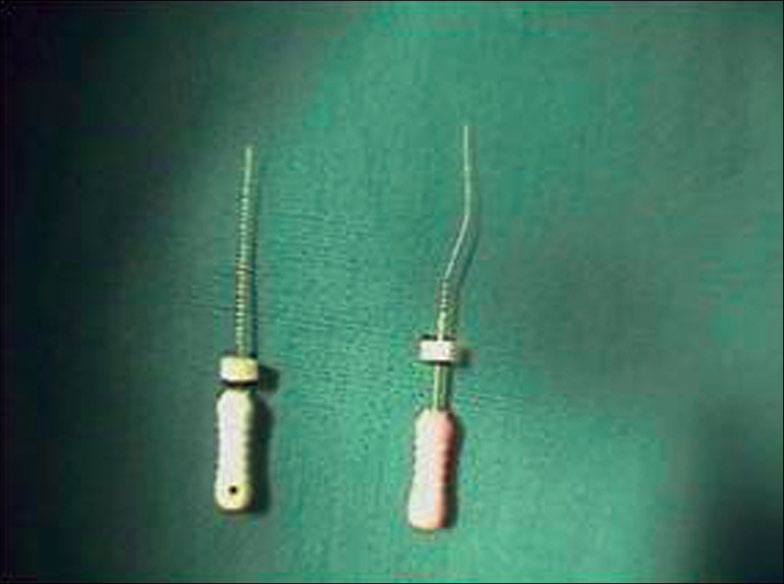
No:10 scouting file to check the patency of the canal

For determining the patency of the root canals, No:10 SS K-files were used [Figure 2]. The buccal canal was easily negotiated to the apex, as it had only one curvature, towards the distal. The buccal canal was cleaned and shaped using Protaper rotary instruments (Sx (Shaper file x), S1 (shaper 1), S2 (shaper 2)). Saline, NaOCl (Sodium hypochlorite), and EDTA (Ethylene diamine tetra acetic acid) were used as irrigants.

**Figure 3:**
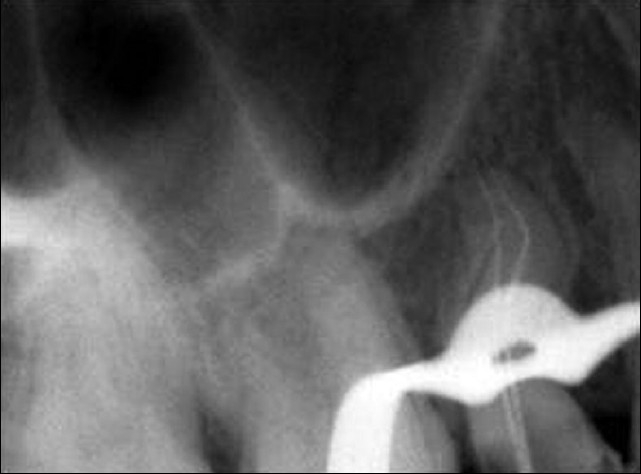
Working length radiograph

**Figure 4:**
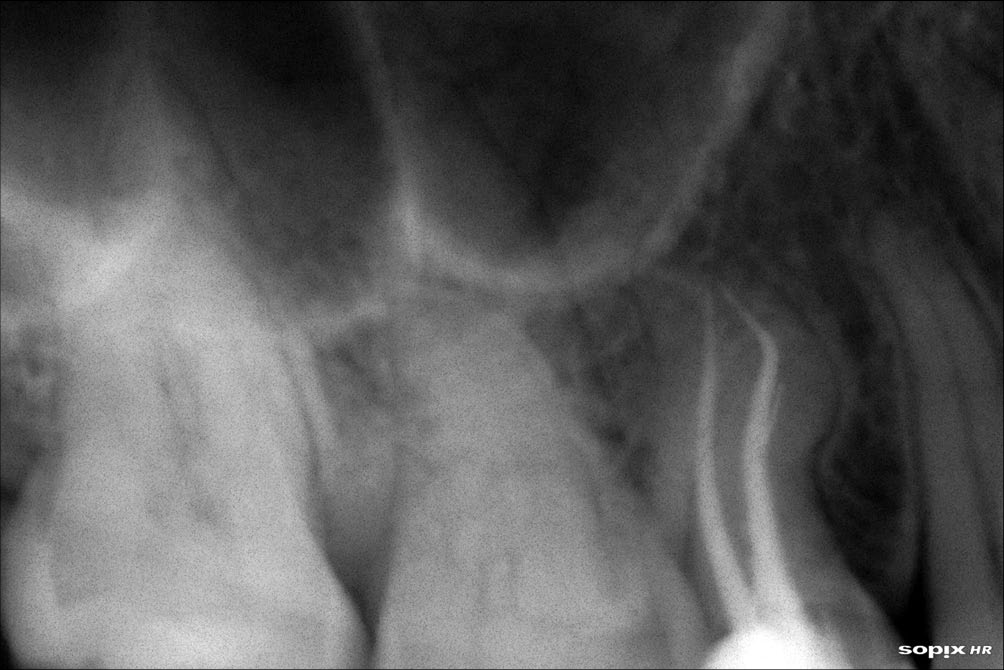
Obturation radiograph

Unlike the buccal root, the palatal root was negotiable only up to the middle third of the root canal. There was resistance to the movement of the patency file. The palatal root canal was enlarged with protaper rotary instruments (Sx) in a crown down motion, right up to the straight portion of the canal. Once this was done, the patency file slid in easily, to the full length. The working length was established using an apex locator (Root ZX, J. Morita, Mfg. Corp, Japan) and confirmed using a radiograph [Figure 3]. The root canal was initially enlarged using NiTi hand files (MANI, Inc, Japan). Three percent NaOCl, EDTA and saline were used as irrigants. The apical portion of the palatal canal was prepared using short amplitude filing. The apical portion and the middle portion were merged using circumferential filing. Both the buccal and palatal canals were obturated using Guttapercha by cold lateral compaction technique [Figure 4].

## DISCUSSION

The ‘S’ shaped canal has two curves, with the apical curve being very difficult to negotiate. The chances of strip perforation are very high in these root canals. (Depending on the degree of the apical curvature, in a few cases it is impossible to instrument this area.) Guttman[4] suggested preflaring the coronal 1/3^rd^ of the canal (at the expense of the tooth structure) to reduce the angle of curvature. Once this is done, it is easy to negotiate the remainder of the root canal.

In this case, both the canals were not S-shaped. The buccal canal had only a mild distal curvature; hence this canal was prepared in a crown-down technique using protaper rotary instrument.

As the palatal canal was doubly curved, a double flare technique was used to enlarge this canal. The access cavity was flared in the coronal-third, in order to reduce the angle of curvature to the first curve. Reducing the angle of curvature by flaring the access (at the expense of the tooth structure) will make the approach to the second curve much easier.[5] Preflaring, in this case, was done with Sx of the protaper instrument. Once this was done, the palatal canal was negotiable, up to the apex. NiTi hand files, with 0.02 taper, were used to prepare the apical portion of the root canal. Short amplitude filing was done to enlarge the apical portion and also to merge it with coronal-third of the root canal. The apical enlargement was limited to size 25 only. Any over enlargement can easily result in perforation in these canals.

## CONCLUSION

Understanding the complex root canal morphology and choosing a canal preparation technique more suited for such morphology, will contribute to successful endodontic treatment.
